# 1*H*-pyrrole-2,5-dione-based small molecule-induced generation of mesenchymal stem cell-derived functional endothelial cells that facilitate rapid endothelialization after vascular injury

**DOI:** 10.1186/s13287-015-0170-6

**Published:** 2015-09-15

**Authors:** Byeong-Wook Song, Il-Kwon Kim, Seahyoung Lee, Eunhyun Choi, Onju Ham, Se-Yeon Lee, Chang Yeon Lee, Jun-Hee Park, Jiyun Lee, Hyang-Hee Seo, Woochul Chang, Cheesoon Yoon, Ki-Chul Hwang

**Affiliations:** EIT/LOFUS R&D Center, International St. Mary’s Hospital, Simgokro 100beongil 25, Incheon, 404-834 Republic of Korea; Institute for Bio-Medical Convergence, College of Medicine, Catholic Kwandong University Gangneung, Beomilro 579beongil 24, Gangwon-do, 210-701 Republic of Korea; Brain Korea 21 PLUS Project for Medical Science, Yonsei University, Yeonsero 50, Seoul, 120-752 Republic of Korea; Department of Integrated Omics for Biomedical Sciences, Yonsei University, Yeonsero 50, Seoul, 120-752 Republic of Korea; Department of Biology Education, Pusan National University, Busandaehakro 63beongil 2, Busan, 609-735 Republic of Korea; Department of Cardiovascular & Thoracic Surgery, College of Medicine, Catholic Kwandong University, Beomilro 579beongil 24, Gangneung, Gangwon-do 210-701 Republic of Korea

## Abstract

**Introduction:**

Despite the success of interventional processes such as drug-eluting stents, complete prevention of restenosis is still hindered by impaired or delayed endothelialization or both. Here, we report that 1*H*-pyrrole-2,5-dione-based small molecule-generated mesenchymal stem cell-derived functional endothelial cells (MDFECs) facilitated rapid transmural coverage of injured blood vessels.

**Methods:**

Small molecules that induced CD31 expression were screened by principal component analysis (PCA). Rat mesenchymal stem cells (MSCs) were treated with selected small molecules for up to 16 days, and the expression levels of CD90 and CD31 were examined by immunocytochemistry. *In vitro* functional assays of MDFECs, including tube formation assays and nitric oxide production assays, were performed. After MDFECs (intravenous, 3×10^6^ cells per animal) were injected into balloon-injured rats, neointima formation was monitored for up to 21 days. The endothelial coverage of denuded blood vessels was evaluated by Evans Blue staining. The functionality of repaired blood vessels was evaluated by measuring vasorelaxation and hemodynamic changes. Additionally, derivatives of the selected small molecules were examined for their ability to induce endothelial markers.

**Results:**

PCA indicated that 3-(2,4-dichlorophenyl)-4-(1-methyl-1*H*-indol-3-yl)-1*H*-pyrrole-2,5-dione effectively induced MDFECs. MDFECs inhibited the neointima formation of denuded blood vessels by facilitating more rapid endothelialization. Further examination indicated that derivatives with a 1H-pyrrole-2,5-dione moiety are important for initiating the endothelial cell differentiation of MSCs.

**Conclusions:**

Small molecules with 1*H*-pyrrole-2,5-dione as a core structure have great potential to improve the efficacy of MSC-based cell therapy for vascular diseases, such as atherosclerosis and restenosis.

**Electronic supplementary material:**

The online version of this article (doi:10.1186/s13287-015-0170-6) contains supplementary material, which is available to authorized users.

## Introduction

Restenosis refers to the recurrence of blood vessel narrowing due to neointima formation, which is characterized by uncontrolled proliferation of vascular smooth muscle cells (VSMCs) [1]. Although the introduction of innovative approaches, such as the drug-eluting stent, has significantly reduced the rate of restenosis [2], complete prevention of restenosis is still not achievable with frequent late stent thrombosis due to impaired or delayed endothelialization or both [3]. The importance of a functional endothelium in preventing intimal thickening and vascular thrombosis has long been recognized [4–6]. Therefore, finding a way to achieve rapid and sufficient transmural coverage with a functional endothelial layer may further improve the outcomes of current interventional approaches for managing atherosclerosis.

During the last decade, MSCs have been the major type of stem cells for therapy because of their self-renewal and multilineage differentiation ability [7], relatively easy isolation protocol from abundant sources [8–10], and low immunogenicity [11, 12]. These characteristics make MSCs the most commonly used stem cells in numerous clinical studies [13]. Previous studies have investigated the feasibility of differentiating MSCs into endothelial cells (ECs) and demonstrated that MSC-derived ECs have the characteristics and functions of ECs. Nevertheless, most of those studies focused on *in vitro* validation of EC-like characteristics [14, 15] or angiogenic or vasculogenic potential or both [16, 17] rather than *in vivo* functionality, such as the endothelialization of denuded blood vessels in animal models. The concept of changing the fate of stem cells by using small molecules was introduced about a decade ago [18], and our group has empirically demonstrated that it is possible to direct cell fate by using various small molecules [19–21].

In this report, we describe the generation of MSC-derived functional ECs (MDFECs) that achieve rapid transmural coverage of injured blood vessels by using 3-(2,4-dichlorophenyl)-4-(1-methyl-1*H*-indol-3-yl)-1*H*-pyrrole-2,5-dione. Further experimental data suggested that the derivatives of this molecule with 1*H*-pyrrole-2,5-dione as a core moiety have a great potential to improve the efficacy of MSC-based cell therapy for vascular diseases, such as atherosclerosis and restenosis.

## Methods

### Principal component analysis

Principal component analysis (PCA) was conducted on the basis of numerical values derived from the sandwich enzyme-linked immunosorbent assay (ELISA). We scaled the two sets of the coordinates to plot them together on the map. The three largest principal components of the PCA analysis are PC1, PC2, and PC3. The detailed procedures were performed as previously described [19].

### Sandwich enzyme-linked immunosorbent assay

Polyvinylchloride microtiter high-binding plates (96-well) were coated overnight with 100 ng of the capture antibody at 4 °C. The plates were washed with phosphate-buffered saline (PBS), and we inhibited the capture antibody with 5 % bovine serum albumin (BSA) in PBS at room temperature, overnight. After the plate was washed with PBS, 5 μg of cell lysate was added to each well with blocking buffer, and the plate was incubated for 90 min at 37 °C. The plate was washed with PBS containing 0.02 % Tween-20 (0.02 % PBS-T). After the detector antibody was added, the plate was incubated for 2 h at room temperature in a humid atmosphere and was washed with 0.02 % PBS-T. Next, the plate was incubated again with a peroxidase-conjugated secondary antibody with 3 % BSA for 90 min at 37 °C and then washed with 0.02 % PBS-T. Finally, 100 μl of tetramethylbenzidine solution (Sigma-Aldrich, St. Louis, MO, USA) was added as a substrate. After 10 min, 25 μl of 0.1 M sulfuric acid was added to stop the reaction, and the absorbance was measured immediately at 450 nm on an ELISA plate reader (Bio-Rad Laboratories, Hercules, CA, USA).

### Animals

Four- and eight-week-old Sprague–Dawley rats were used for MSC isolation and the vascular injury model, respectively. Before the experimental procedure, the rats were anesthetized with zoletil (20 mg/kg) and xylazine (5 mg/kg). All animal experimental procedures were approved by the Institutional Animal Care and Use Committee of Yonsei University College of Medicine in cooperation with the Association for Assessment and Accreditation of Laboratory Animal Care and performed in accordance with the Guidelines and Regulations for Animal Care.

### Isolation and culture of rat MSCs

Bone marrow-derived MSCs were isolated and collected from aspirates of rat femurs and tibias with 10 ml of MSC medium consisting of Dulbecco’s modified Eagle’s medium (DMEM)-low glucose supplemented with 10 % fetal bovine serum (FBS) (Invitrogen) and 1 % antibiotic-penicillin and streptomycin. Mononuclear cells that had been recovered from the interface of Ficoll-Paque PLUS (GE Healthcare, Little Chalfont, UK)-separated bone marrow were washed twice and resuspended in DMEM with 10 % FBS and then plated at a density of 1×10^6^ cells per 100-mm dish. The cultures were maintained at 37 °C in a humidified atmosphere containing 5 % CO_2_. After 72 h, the non-adherent cells were discarded, and the adherent cells were thoroughly washed twice with PBS. Fresh MesenPRO RS™ Medium (Invitrogen) was added and then replaced every 3 days for approximately 10 days to achieve stable multi-potentiality. The characterization of the MSCs isolated by using our in-house protocol has been previously described [22], and additional experiments for evaluating the differentiation potential of the MSCs used in the present study confirmed their osteogenic and adipogenic differentiation potential (Additional file 1: Figure S1).

### *In vitro* differentiation assay

Isolated MSCs were subjected to differentiation assays by using the rat MSC functional identification kit (SC020; R&D Systems, Minneapolis, MN, USA) in accordance with the protocols of the manufacturer.

### Treatment of small molecules

At passage 1 or 2, MSCs were seeded in 60-mm dishes at 1×10^5^ cells/ml and treated with a final concentration of 1 μM of small molecules, including SB216763 (EMD Millipore, Billerica, MA, USA) and SB derivatives (Sigma-Aldrich; Santa Cruz Biotechnology, Dallas, TX, USA; and JINC). The media (DMEM with 10 % FBS) were replaced with fresh small molecule-containing media every 3 days for 16 days.

### Reverse transcription-polymerase chain reaction analysis

The expression levels of various genes were analyzed by reverse transcription-polymerase chain reaction (RT-PCR). Total RNA was prepared by using the UltraspectTM-II RNA system (Biotecx Laboratories, Inc., Houston, TX, USA), and single-stranded cDNA was then synthesized from the isolated total RNA by using avian myeloblastosis virus (AMV) reverse transcriptase. A 20-μl reverse transcription reaction mixture containing 1 μl of total RNA, 1X reverse transcription buffer (10 mM Tris–HCl, pH 9.0, 50 mM KCl, and 0.1 % Triton X-100), 1 mM deoxynucleoside triphosphates (dNTPs) 0.5 units of RNase inhibitor, 0.5 μg of oligo(dT)_15_, and 15 units of AMV reverse transcriptase was incubated at 42 °C for 15 min, heated to 99 °C for 5 min, and then incubated at 4 °C for 5 min. PCR was performed for 35 cycles with 3′ and 5′ primers based on the sequences of various genes. The primers are listed in the Additional file 2: Table S1.

### Immunocytochemistry

Cells were grown on four-well plastic dishes. After incubation, the cells were washed twice with PBS and then fixed with 4 % paraformaldehyde in PBS for 30 min at room temperature. The cells were washed again with PBS and then permeabilized for 30 min in PBS containing 0.2 % Triton. Next, the cells were blocked in PBS containing 10 % goat serum and incubated for 1 h with CD90, CD31, vascular endothelial growth factor (VEGF) receptor 1 (Flk-1), β-catenin (Santa Cruz Biotechnology, 1:200), and acetylated α-tubulin (Abcam, Cambridge, MA, USA, 1:200). The cells were washed again three times for 10 min with PBS and incubated with a FITC (fluorescein isothiocyanate)-conjugated secondary antibody (Jackson ImmunoResearch Laboratories, Inc., West Grove, PA, USA, 1:500) for 1 h. Finally, the cells were treated with DAPI (4′,6-diamidino-2-phenylindole) (Sigma-Aldrich) to stain nuclei for 2 min and then mounted on slides. Photographs of the cells were acquired by using an immunofluorescence microscope (Carl Zeiss, Oberkochen, Germany, LSM700). All images were acquired by using an excitation filter with a reflected light fluorescence microscope and transferred to a computer equipped with ZEN software (Carl Zeiss).

### Lipid uptake assay using DiI-LDL

A lipid uptake assay using DiI-LDL (3,3′-dioctadecylindocarbocyanine-low density lipoprotein) was conducted. The cells were incubated with DiI-LDL (10 μg/ml) for 4 h at 37 °C. The cells were lysed in 0.1 N NaOH and 0.1 % SDS and shaken for 10 min followed by fluorescence reading for DiI-LDL (excitation/emission at 530/580 nm). The fluorescence of DiI-LDL was normalized by the cell lysate protein concentrations as previously described [23].

### Nitric oxide production assay

In brief, the cells were washed with warm PBS and stimulated with 5 μM acetylcholine (ACh) in phenol red-free DMEM for 60 min. The media were collected and spun at 2000*g* for 1 min before being transferred to a new tube and subjected to a nitric oxide (NO) production assay. We followed the protocol included with the NO release Fluorometric Assay Kit (BioVision, Milpitas, CA, USA).

### Fluorescence *in situ* hybridization analysis

Arterial sections (3 μm) were mounted on gelatin-coated glass slides to ensure different stains. After de-paraffinization and re-hydration, we used STAR*FISH^©^ Rat 12/Y Paints (Cambio,) as described in the protocol of the manufacturer.

### Evans Blue staining and morphometric analysis

Femoral vein injections with 5 % Evans Blue dye (Sigma-Aldrich) were performed 60 min before sacrifice to analyze the denuded and recovered areas. The harvested common carotid artery was fixed with 10 % formalin, paraffin-sectioned cross-sectionally, and stained with hematoxylin and eosin. Re-endothelialization and the intima/media area were measured by using ImageJ software (National Institutes of Health, Bethesda, MD, USA).

### Vascular injury and cell injection

A 2-Fr Fogarty arterial embolectomy catheter (Edwards Lifesciences, Irvine, CA, USA) was inserted in the left common carotid artery of the rats. The balloon was inflated in the common carotid artery and moved back and forth three times for endothelial denudation. For the delivery of MSCs treated with SB for 16 days, 3×10^6^ cells in 0.3 ml of saline were injected per animal via intravenous (i.v.) injection in the femoral vein. For the MSC control group, MSCs without SB treatment were delivered (3×10^6^ cells per 0.3 ml saline, per animal) via i.v. injection through the femoral vein. Animals were sacrificed 3, 5, 7, 14, and 21 days after the balloon injury (BI) for further analysis. The *in vivo* studies were conducted by using three independent animals (rather than three animals of one cohort).

### Aortic ring preparation and vasodilator responsiveness

After anesthesia, the common carotid arteries were excised and placed in HEPES-Tyrode’s solution (10 mM glucose, 10 mM HEPES, 134 mM NaCl, 5.6 mM KCl, 1 mM MgCl_2_, and 2.5 mM CaCl_2_), which was aerated with 100 % O_2_. The arteries were prepared as ring segments (3 mm in length). Each artery ring was mounted horizontally between two parallel stainless steel hooks in a temperature-controlled 3-ml organ bath. One hook was fixed, whereas the other was connected to a force transducer (UFER; Kishimoto Medical Instruments, Kyoto, Japan) to measure isometric contraction. After an equilibration period of 30 min at 37 °C in HEPES-Tyrode’s solution aerated continuously by 100 % O_2_, the ring segments were stretched passively by imposing an optimal resisting force of 10 mN, which was found to be the optimal force for use with the 70 mM high K^+^ HEPES-Tyrode’s solution (K^+^ substitution for Na^+^). The arterial rings were repeatedly contracted with 70 mM high K^+^ HEPES-Tyrode’s solution until stable responses were obtained. Submaximal contraction was elicited by 50 mM high K^+^ HEPES-Tyrode’s (50 K) solution. Endothelial-dependent vasodilation was induced by the addition of progressive doses of ACh (Sigma-Aldrich; 10^−8^-10^−4^ M).

### *In vivo* blood flow measurement

After the rats were anesthetized, a small incision was carefully made in the throat area to isolate the left common carotid artery. A transit time perivascular flow meter (T402; Transonic System Inc., Ithaca, NY, USA) and a transonic flow probe (1.0PRB4284) were used to measure the blood flow. The equipment was calibrated by using a standard flow meter in milliliters per minute. The waveform of the blood flow was recorded for 10 min and analyzed by using LabChart 7 software (ADInstruments, Dunedin, New Zealand).

### Western blot

Western blot was performed by using primary antibodies against phosphorylated glycogen synthase kinase 3 beta (GSK3β) at Ser9, phosphorylated β-catenin, and β-actin (Santa Cruz Biotechnology, 1:1000).

### *In vitro* angiogenesis

Analysis of capillary formation was performed using an *in vitro* angiogenesis kit (Chemicon International Inc., Billerica, MA, USA) in accordance with the instructions of the manufacturer. A gel matrix solution (50 μl) was applied to each well of a 96-well plate, which was incubated for 1 h at 37 °C. The cells were then trypsinized and resuspended at a density of 5×10^3^ cells in 50 μl of DMEM with or without VEGF (20 nM), plated on the gel matrix, and incubated for 24 h. The total length of the formed tube was calculated by using the ImageJ program from three independent experiments.

### Scanning electron microscopy

After fixation with glutaraldehyde, the slides were washed for 5 min in malonic sodium phosphate buffer (pH 7.3). The specimens were fixed in 1 % osmium tetroxide for 2 h at 4 °C. The samples were separately washed for 5 min in water, 50 % ethanol, and 70 % ethanol; for 15 min in 95 % ethanol; and two times for 15 min each in absolute ethanol. An SEM S-800 (Hitachi, Tokyo, Japan) was used with magnifications of 10 to 10,000.

### Statistical analysis

Quantitative data are expressed as the mean ± standard deviation of at least three independent experiments. For statistical analysis, one-way analysis of variance with Bonferroni correction was performed by using OriginPro 8 SR4 software (version 8.0951; OriginLab Corporation, Northampton, MA, USA). Data normality was tested by Shapiro-Wilk test. A *P* value of less than 0.05 was considered statistically significant.

## Results and Discussion

In a preliminary screening to identify small molecules that induce the EC differentiation of MSCs, we treated MSCs with commercially available small-molecule inhibitors of six different groups of the protein kinase superfamily—kinase group AGC (protein kinase A (PKA), protein kinase C (PKC), and protein kinase G (PKG)), CaM (calcium/calmodulin-dependent protein kinase), CK1 (casein kinase 1 group), kinase group CMGC (cyclin-dependent kinase (CDK), mitogen-activated protein kinase (MAPK), GSK3, and CDC-like kinase (CLK kinase)), TK (tyrosine kinase), and TKL (tyrosine-kinase like group) kinase—every 3 days (each, 1 μM) for up to 16 days and evaluated the differentiation status by sandwich ELISA for the endothelial marker CD31. Subsequent PCA on the profile matrix for the cross-relationship between specific cell types and small molecules indicated that 3-(2,4-dichlorophenyl)-4-(1-methyl-1H-indol-3-yl)-1H-pyrrole-2,5-dione, known as SB216763 (SB), was a hit compound that induces the EC differentiation of MSCs (Additional file 3: Figure S2). Furthermore, compared with untreated MSCs, SB-treated MSCs expressed higher levels of CD34 and von Willebrand Factor (vWF) (Additional file 4: Figure S3). Although SB induced the expression of EC markers, such as CD31 and vWF, this induction does not necessarily guarantee that those cells possess EC-related functionality. Therefore, we conducted additional tests, including tests to assess the EC functionality of SB-treated cells, and we refer to those cells as induced MDFECs (iMDFECs).

**Fig. 1:**
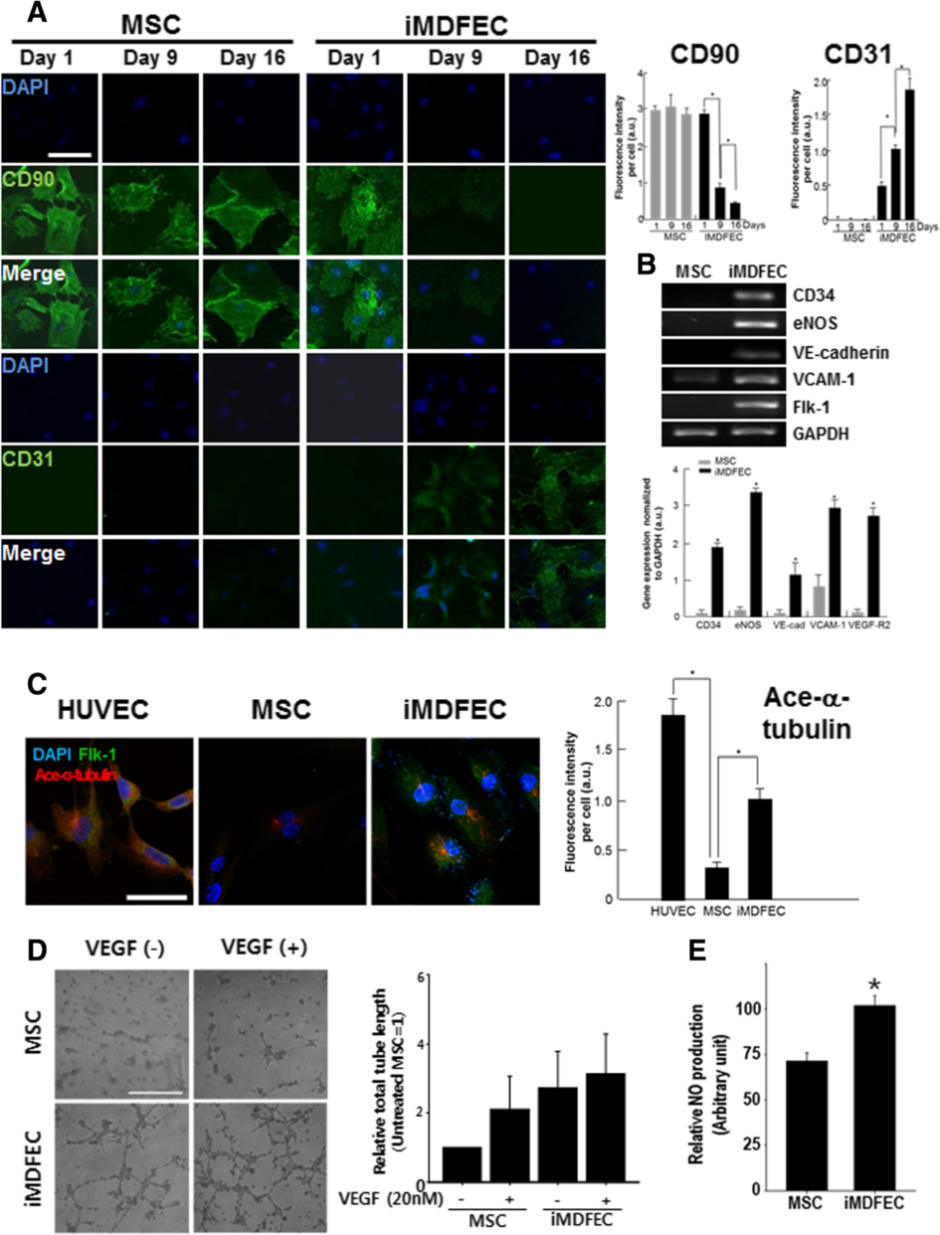
Small molecule-treated MSCs (iMDFECs) change cell type-specific marker expression and gain angiogenic ability *in vitro.* **a** Immunocytochemical monitoring of MSCs treated with SB (1 μM, every 3 days). CD90 and CD31 were used as representative markers of MSCs and ECs, respectively. Scale bar = 50 μm. Fluorescence intensities are quantified on the *upper right side*. *P < 0.05. **b** EC marker gene expression in iMDFECs. mRNA expression levels of CD34, eNOS, VE-cadherin, VCAM-1, and Flk-1 were measured by using reverse transcription-polymerase chain reaction. GAPDH was used for normalization. *P < 0.05. **c** Flk-1 and Ace-α-tubulin expressions in iMDFECs. *P < 0.05. **d** After 16 days of treatment, iMDFECs were re-seeded and stimulated with vascular endothelial growth factor (VEGF) (20 nM) for 24 h. The angiogenic ability (tube formation; total tube length) of iMDFECs was compared with that of control MSCs. Scale bar = 400 μm. *P < 0.05. **e** Cells were stimulated with 5 μM acetylcholine for 60 min, and the media were collected and subjected to an NO release fluorometric assay. Data represent mean ± standard deviation of at least three independent experiments. *EC* endothelial cell, *eNOS* endothelial nitric oxide synthase, *Flk-1* vascular endothelial growth factor receptor 1, *GAPDH* glyceraldehyde 3-phosphate dehydrogenase, *iMDFEC* induced mesenchymal stem cell-derived functional endothelial cell, *MSC* mesenchymal stem cell, *NO* nitric oxide, *SB* SB216763, *VCAM-1* vascular cell adhesion molecule 1, *VE-cadherin* vascular endothelial cadherin

Morphological examination revealed that, although the iMDFECs did not show drastic morphological changes (Additional file 5: Figure S4A), they had a time-dependent decrease in the mRNA expression of the MSC marker CD71 (Additional file 5: Figure S4B) and an increase in CD31 mRNA expression (Additional file 5: Figure S4C). Immunocytochemical staining for CD90 and CD31 also indicated that the CD31 expression in iMDFECs increased but that the expression of the MSC marker CD90 decreased with time (Fig. 1a). Furthermore, the iMDFECs showed increased expression levels of other EC markers, such as CD34, endothelial nitric oxide synthase (eNOS), vascular endothelial cadherin (VE-cadherin), vascular cell adhesion molecule 1 (VCAM-1), and Flk-1 (Fig. 1b and c). Because adhesion molecules, such as VE-cadherin and VCAM-1, are important players in mediating EC adhesion and the subsequent blood vessel maturation [24, 25], the increased expression levels of these molecules are expected to play a crucial role in the initial settlement of iMDFECs in damaged blood vessels in the case of systemic infusion for therapeutic purposes (i.e., re-endothelialization of denuded blood vessels). Additionally, when stimulated with VEGF (20 nM), iMDFECs showed a trend of increased total tube length compared with that of the control MSCs, suggesting that the iMDFECs were responsive to angiogenic growth factor (Fig. 1d). Furthermore, compared with control MSCs, iMDFECs produced a higher amount of NO (71.50±4.55 vs. 101.75±5.63) in response to ACh (Fig. 1e), which is known to induce NO release from ECs [26]. A lipid uptake assay using DiI-LDL indicated increased lipid uptake by iMDFECs compared with that of untreated MSCs (Additional file 6: Figure S5).

**Fig. 2:**
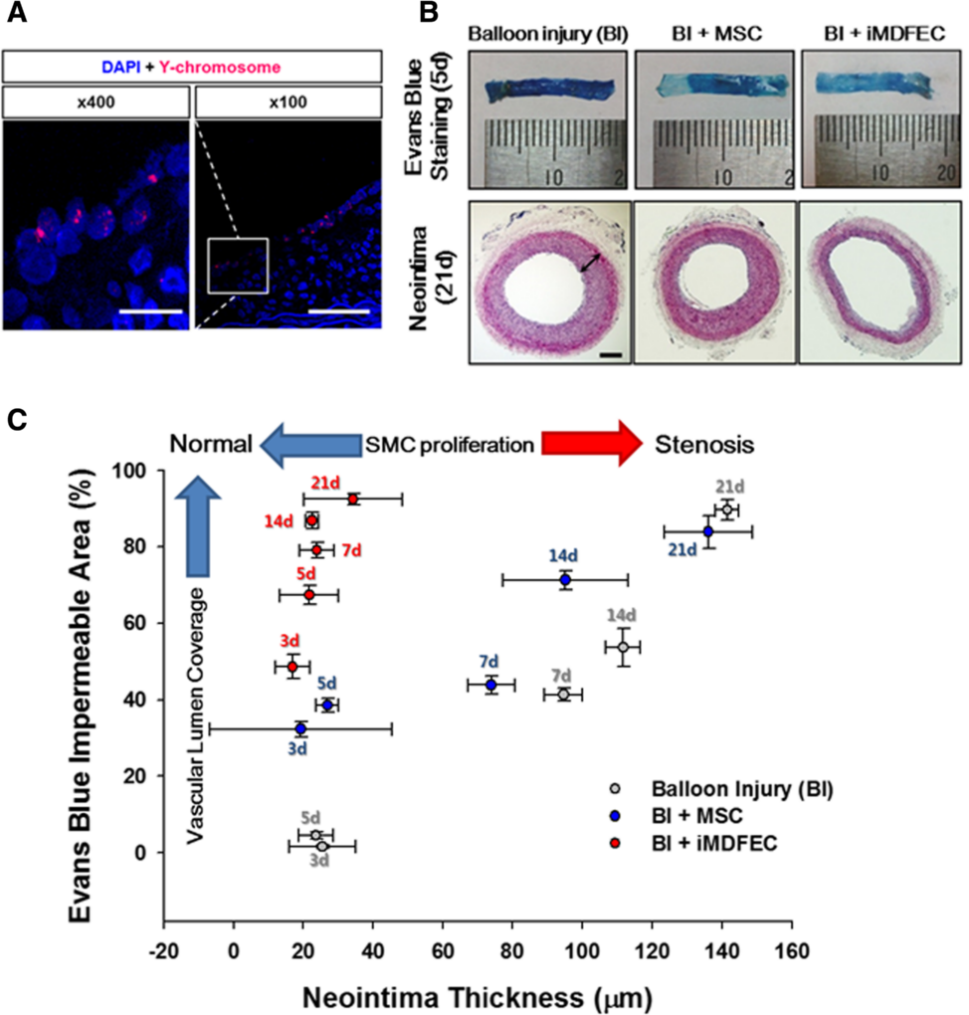
Transplantation of iMDFECs after vascular injury significantly inhibited neointima formation and achieved rapid endothelialization of denuded vessels *in vivo*. **a** Tracking transplanted iMDFECs by using Y chromosome staining (fluorescence *in situ* hybridization). Left scale bar = 12.5 μm. Right scale bar = 50 μm. **b** Representative images of an Evans Blue-stained carotid artery showing transmural coverage (*upper panels*) and a cross-section of a hematoxylin-and-eosin-stained carotid artery to show neointima formation (*bottom panels*). More blue indicates increased permeability. Scale bar = 200 μm. **c** Time-dependent changes in transluminal coverage were plotted against neointima thickness (n = 3). See Additional file 9: Figure S8 for statistical significance. *DAPI* 4ʹ,6-diamidino-2-phenylindole, *iMDFEC* induced mesenchymal stem cell-derived functional endothelial cell, *MSC* mesenchymal stem cell, *SMC* smooth muscle cell

To assess the feasibility of using iMDFECs in therapeutic approaches and to evaluate *in vivo* functionality, we systemically transplanted control MSCs or iMDFECs in vascularly injured animals via i.v. injection (3×10^6^ cells per 0.3 ml of saline, per animal) through the femoral vein. We used male rats and female rats as the source of MSCs and for the vascular injured animal model, respectively. Thus, we could track the transplanted cells by detecting Y chromosomes. Immunohistochemical staining of the carotid artery for Y chromosomes demonstrated that some of the infused iMDFECs resided in the innermost layer of the carotid artery, suggesting that iMDFECs physically contributed to the formation of a functional endothelial layer (Fig. 2a). The relative amount of cells incorporated into the injured blood vessel was higher in the iMDFEC-transplanted group than in the MSC-transplanted control group (Additional file 7: Figure S6).

The results of Evans Blue staining to evaluate the permeability of the carotid artery indicated that, although there was no significant difference in the transluminal coverage among the groups after 3 weeks (BI vs. BI + MSC vs. BI + iMDFEC group, 89.59±2.66 vs. 83.87±4.26 vs. 92.47±1.47, respectively), the iMDFEC-transplanted group showed a rapid increase in the dye-impermeable area during the first week after vascular injury compared with that of other groups (79.04±1.95 vs. the BI and BI + MSC group values of 41.31±1.75 and 43.86±2.34, respectively) (Fig. 2b and Additional file 8: Figure S7). The Evans Blue-impermeable area of the BI + iMDFEC group was significantly higher than those of the BI and BI + MSC groups from day 3 to 14 (Additional file 9: Figure S8A), suggesting that relatively faster re-endothelialization occurred with iMDFEC transplantation. Furthermore, neointima formation was significantly inhibited in the iMDFEC-transplanted group compared with the other groups on day 21 (34.22±14.09 compared with 136.13±12.71 for the BI + MSC group and 141±3.37 for the BI-only group) (Fig. 2c). The neointima thickness of the BI + iMDFEC group was significantly lower than that of the other groups from day 7 to 21 (Additional file 9: Figure S8B). These data together indicate that iMDFECs facilitated relatively faster endothelialization of denuded blood vessels, and this change, in turn, suppressed neointima formation, recapitulating the role of a functional endothelial layer in preventing aberrant outgrowth of underlying VSMCs [27].

**Fig. 3:**
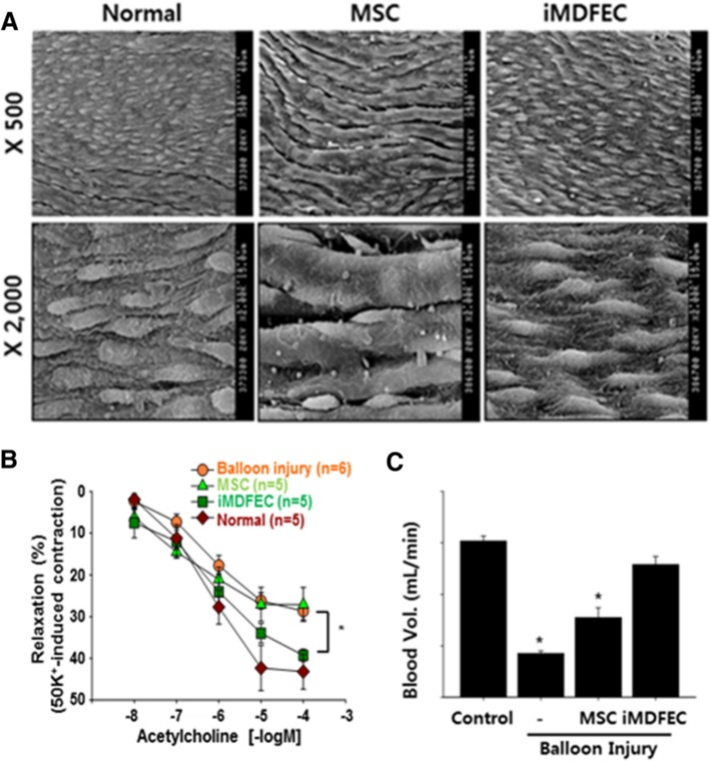
The morphology and function of the iMDFEC-transplanted carotid artery have greater similarity to those of intact blood vessels compared with those of the MSC-transplanted carotid artery. **a** The innermost layer of explanted carotid arteries was examined by using a scanning electron microscope. **b** Relaxation profiles of acetylcholine-stimulated carotid artery segments. The acetylcholine-induced relaxation responses are expressed as the percentage of the maximal contractions in response to 50 K^+^ solution. *P < 0.05. **c** Blood flow was measured for each group. *P < 0.05 compared with the control. *G-MSC* gingival mesenchymal stem cell, *iMDFEC* induced mesenchymal stem cell-derived functional endothelial cell, *MSC* mesenchymal stem cell

Closer examination using a scanning electron microscope indicated that the morphology of the innermost layer of the carotid artery of iMDFEC-transplanted animals resembled that of a control, uninjured endothelium but that the innermost layer of the carotid artery from the MSC-transplanted animals showed a less dense organization of cells with relatively bigger gaps between cells (Fig. 3a). Bone marrow-derived endothelial-like cells are capable of vasorelaxation in response to ACh treatment [28, 29]. We also examined the ACh-induced vasorelaxation of carotid arteries from iMDFEC-transplanted animals. Our data indicate that the vasorelaxation of carotid arteries of the iMDFEC-transplanted group was substantially higher than that of the control MSC-transplanted group (43.42±4.46 vs. 27.10±4.06), and this value was comparable to that of normal carotid arteries (Fig. 3b). Furthermore, the iMDFEC-infused group had the highest flow rate (3.89±0.22 ml/min) compared with that of both the sham group (1.40±0.09 ml/min) and the control MSC-infused group (2.22±0.10 ml/min) (Fig. 3c). Taken together, these data indicate that the carotid artery from the animals that received iMDFEC infusion was functionally comparable to that of a normal carotid artery and was much improved compared with that from the animals that received MSC infusion.

SB is a GSK3β inhibitor [30], and GSK3β is known to inhibit the Wnt/β-catenin signaling pathway by facilitating phosphorylation of β-catenin at Ser33, Ser37, and Thr41 [31]; this phosphorylation initiates ubiquitination and proteasomal degradation of cytoplasmic β-catenin [32]. In fact, a GSK3β inhibitor has been reported to promote activation of the Wnt/β-catenin signaling pathway [33]. Our results also indicate that SB treatment increased the phosphorylation of GSK3β at Ser9 (Additional file 10: Figure S9A), which is known to inactivate GSK3β [34], and increased the nuclear localization of β-catenin (Additional file 10: Figure S9B). Nevertheless, the EC differentiation-inducing ability of SB may not be due primarily to its inhibitory action on GSK3β under our experimental conditions because 3 l additional GSK3β inhibitors with comparable IC_50_ (half maximal inhibitory concentration) values (TWS119, kenpaullone, and indirubin-3′-oxime) [35] showed far inferior potency to induce CD31 expression in MSCs compared with that of SB (Additional file 10: Figure S9C). In other words, the EC differentiation potency of SB may stem from its core structure rather than from its inhibitory effect on GSK3β.

To test this assumption, first, we selected two core structures from SB, 1*H*-pyrrole-2,5-dione and 1*H*-indole, that are expected to exhibit a wide range of biological activities. Based on these structures, we searched commercially available compounds with no confirmed GSK3β inhibitory effects (11 derivatives with a 1*H*-pyrrole-2,5-dione moiety and three derivatives with a 1*H*-indole moiety as the core structure, Additional file 11: Figure S10), and we screened these 14 SB derivatives for their EC differentiation potency by examining the expression levels of the EC markers CD31, vWF, and VCAM-1 after 9 days of treatment.

**Fig. 4:**
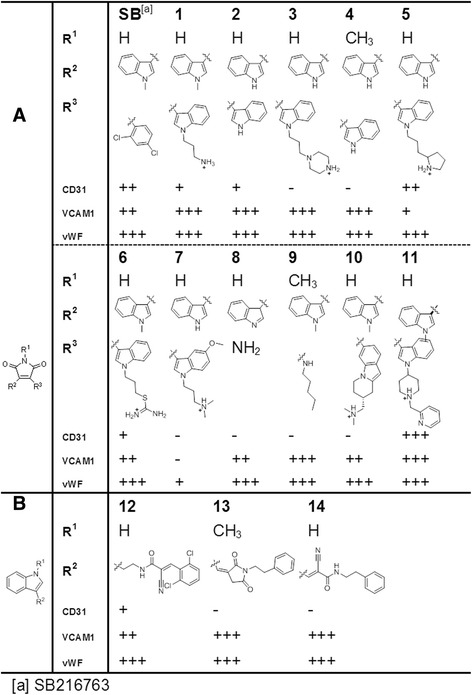
SB derivatives tested for induction of endothelial cell markers in mesenchymal stem cells. **a** Derivatives with a 1*H*-pyrrole-2,5-dione moiety. **b** Derivatives with a 1H-indole moiety. The number of plus signs indicates the number visible polymerase chain reaction bands detected from three independent sets. Minus signs indicate no visible band detected. *SB* SB216763, *VCAM-1* vascular cell adhesion molecule 1, *vWF* von Willebrand factor

The results of RT-PCR screening indicated that five of 11 derivatives with a 1*H*-pyrrole-2,5-dione structure induced the expression of all three EC markers at least once in three independent differentiation tests but that one of three derivatives with a 1H-indole structure did so (Fig. 4), indicating that the 1*H*-pyrrole-2,5-dione structure may be important for the EC differentiating capability of those derivatives. We are currently trying to verify the speculated EC differentiation-inducing effect of 1*H*-pyrrole-2,5-dione and to investigate the possible underlying mechanisms, such as transcription factor regulation, of the 1*H*-pyrrole-2,5-dione effect on EC differentiation. We also plan to synthesize a new series of 1*H*-pyrrole-2,5-dione derivatives to optimize and improve the activity and efficiency of EC differentiation without undesirable effects.

Previous studies have reported the EC differentiation of MSCs by using various methods, including a combination of a mechanical stimulus, such as shear stress, and VEGF [36], plating cell density [37], and the addition of extracellular matrix [17]. Furthermore, more recent studies used small molecules to induce EC differentiation [38, 39]. We also used small molecules to induce the EC differentiation of MSCs, demonstrating that small molecules may be used to induce the EC differentiation of stem cells. Although most of the previous studies focused on an *in vitro* characterization of the differentiated cells, we conducted *in vivo* experiments by using an animal model to examine the functions of differentiated cells. The results of our study demonstrate that the iMDFECs generated by using the identified small molecules are effective in rapid repairing of injured blood vessels *in vivo*.

## Conclusions

In the present study, we conducted PCA by using the results from cell-based chemical screening to identify a small-molecule inducer of the EC differentiation of MSCs and demonstrated that 1*H*-pyrrole-2,5-dione-based small molecules may promote the EC differentiation of MSCs. Our data indicated that the resulting iMDFECs possessed characteristics of ECs *in vitro* and *in vivo*. More significantly, compared with control MSCs, iMDFECs achieved more rapid endothelialization of denuded blood vessels. This iMDFEC-mediated rapid endothelialization was associated with significant inhibition of neointima formation after vascular injury in animals. In addition, the carotid artery of the iMDFEC-infused animals showed much improved functionality, such as blood vessel relaxation and blood flow rate, compared with that of control MSC-infused animals. Taken together, the results show that 1*H*-pyrrole-2,5-dione-based small molecules are potent inducers of MDFECs that can further improve the outcomes of current interventional approaches for managing vascular diseases, such as restenosis and atherosclerosis.

## Additional files

Additional file 1: Figure S1. Differentiation potential of rat MSCs isolated by using an in-house protocol. (**A**) Osteogenic differentiation of MSCs was detected by immunocytochemistry by using anti-osteocalcin antibodies. (**B**) Adipogenic differentiation of MSCs was detected by immunocytochemistry by using anti-FABP4 (fatty acid-binding protein 4) antibodies. (**C**) Chondrogenic differentiation of MSCs was detected by immunocytochemistry by using anti-aggrecan antibodies. *MSC* mesenchymal stem cell. (TIFF 418 kb) Additional file 2: Table S1. Primers for reverse transcription-polymerase chain reaction. (TIFF 62 kb) Additional file 3: Figure S2. Principal component analysis (PCA) suggests that the small molecule KI-7 will induce the EC differentiation of MSCs. The PCA results showing a cross-relationship between specific cell types and the screened small molecules (yellow balls). Chemical names with targets in parenthesis: KI-0: no inhibitor; KI-1: lavendustin (5-(N-2,5-dihydroxybenzyl) aminosalicylic acid (CaMKII); KI-2:(4-(4-(2,3-dihydrobenzo [1, 4] dioxin-6-yl)-5-pyridin-2-yl-1H-imidazol-2-yl)benzamide (CKI); KI-3: 6-cyclohexylmethoxy-2-(4-sulfamoylanilino) purine (CDK1,2); KI-4: 3-(pyridin-2-yl)-4-(4-quinonyl)]-1Hpyrazole (TGFβRI kinase); KI-5: N-[2-((p-bromocinnamyl) amino)ethyl]-5-isoquinolinesulfonamide, 2HCl (PKA); KI-6: (2-[1-(3-dimethylaminopropyl)-5-methoxyindol-3-yl]-3-(1H-indol-3-yl)) maleimide (PKC); KI-7: 3-(2,4-dichlorophenyl)-4-(1-methyl-1H-indol-3-yl)-1H-pyrrole-2,5-dione (GSK3β); KI-8: 4-[(3-bromophenyl) amino]-6,7-diethoxyquinazoline (PTK); KI-9: N- (4-pyridyl)-N-(2,4,6-trichlorophenyl) urea (ROCK); KI-10: 4,5-dimethoxy-2-nitrobenzaldehyde (DNA-PK); KI-11: 4-(4-fluorophenyl)-2-(4-hydroxyphenyl)-5-(4-pyridyl)1H-imidazole (p38 MAPK). *CaMK* calcium/calmodulin-dependent protein kinase; *CDK* cyclin-dependent kinase, *CK* casein kinase I, *DNA-PK* DNA-dependent protein kinase, *GSK* glycogen synthase kinase, *KI* kinase inhibitor, *MAPK* mitogen-activated protein kinase, *MSC* mesenchymal stem cell, *PC* principal component, *PKA* protein kinase A, *PKC* protein kinase C, *PTK* protein tyrosine kinase, *ROCK* rho-associated protein kinase, *TGFβ RI* transforming growth factor beta type I receptor. (TIFF 91 kb) Additional file 4: Figure S3. EC marker expression in iMDFECs. MSCs treated with SB for 16 days to induce EC differentiation. **(A)** CD34 expression was measured by immunocytochemistry by using anti-CD34 antibodies. **(B)** vWF expression was measured by immunocytochemistry by using anti-vWF antibodies. *EC* endothelial cell, *iMDFEC* induced mesenchymal stem cell-derived functional endothelial cell, *MSC* mesenchymal stem cell, *SB* SB216763, *vWF* von Willebrand factor. (TIFF 269 kb) Additional file 5: Figure S4. Time-dependent changes of iMDFECs. **(A)** Morphological examination of iMDFECs at indicated time points. **(B)** The mRNA expression levels of the MSC marker CD71 and the EC marker CD31 were examined at the indicated time points. Data represent the mean ± standard deviation of at least three independent experiments. *EC* endothelial cell, *iMDFEC* induced mesenchymal stem cell-derived functional endothelial cell, *MSC* mesenchymal stem cell. (TIFF 270 kb) Additional file 6: Figure S5. Lipid uptake assay using DiI-LDL. MSCs or iMDFECs were incubated with DiI-LDL (10 μg/ml) for 4 h. The cells were lysed in 0.1 N NaOH and 0.1 % SDS, and the amount of DiI-LDL was determined by fluorescence reading (excitation/emission at 530/580 nm). The fluorescence of DiI-LDL was normalized by the cell lysate protein concentrations. *DiI-LDL* 3,3′-dioctadecylindocarbocyanine-low density lipoprotein, *iMDFEC* induced mesenchymal stem cell-derived functional endothelial cell, *MSC* mesenchymal stem cell. (TIFF 33 kb) Additional file 7: Figure S6. Evaluation of amount of MSCs or iMDFECs incorporated by using SRY as a marker of incorporated male-origin transplanted cells. Total RNA was prepared from the common carotid artery harvested at day 21 after the balloon injury. Data represent the mean ± standard deviation of three independent experiments. *iMDFEC* induced mesenchymal stem cell-derived functional endothelial cell, *MSC* mesenchymal stem cell. (TIFF 114 kb) Additional file 8: Figure S7. Time-dependent changes in vessel permeability and neointima thickness after transplantation of MSCs/MDFECs in balloon-injured animals. **(A)** Images of an Evans Blue-stained carotid artery showing transmural coverage. More blue indicates increased permeability. **(B)** Images of neointima formation at different time points. Scale bar = 200 μm. *MDFEC* mesenchymal stem cell-derived functional endothelial cell, *MSC* mesenchymal stem cell. (TIFF 369 kb) Additional file 9: Figure S8. Quantification of time-dependent changes in neointima thickness and vessel permeability after transplantation of MSCs/iMDFECs in balloon-injured animals. **(A)** Time-dependent changes in the Evans Blue-impermeable area after balloon injury were measured. **(B)** Time-dependent changes in the neointima thickness after balloon injury were evaluated. **P* < 0.05. *iMDFEC* induced mesenchymal stem cell-derived functional endothelial cell, *MSC* mesenchymal stem cell. (TIFF 73 kb) Additional file 10: Figure S9. Effect of SB on β-catenin activation and EC differentiation and efficacy of different GSK3β inhibitors. **(A)** The expression levels of phosphorylated GSK3β (pser9GSK3β) and β-catenin (pβ-catenin) and **(B)** the nuclear localization of β-catenin were examined after MSCs were treated with 1 μM SB216763 for 24 h. Scale bar = 50 μm. *White arrows* indicate nuclear translocated β-catenin. **(C)** Effect of different GSK3β inhibitors (TWS119, kenpaullone, and indirubin-3′-oxime) on the CD31 induction of MSCs. MSCs were treated with different GSK3β inhibitors with varying concentrations (0.1, 0.5, and 1 μM) for 16 days, and the expression of CD31 was examined. *EC* endothelial cell, *GSK3β* glycogen synthase kinase 3 beta, *MSC* mesenchymal stem cell, *SB* SB216763. (TIFF 112 kb) Additional file 11: Figure S10. 1H-pyrrole-2,5-dione moiety is important to induce EC-like differentiation of MSCs. Screening of SB216763 derivatives for inducing EC marker expressions was conducted. To examine the potency of SB216763 derivatives for inducing EC differentiation of MSCs, the MSCs were treated 1 μM of each derivatives for 9 days (media containing corresponding small molecules were changed every 3 days), and the expressions of EC markers CD31, VCAM1, and vWF were examined by reverse transcription-polymerase chain reaction. Samples were collected at 9 days after the initial treatment. *EC* endothelial cell, *MSC* mesenchymal stem cell, *VCAM-1* vascular cell adhesion molecule 1, *vWF* von Willebrand factor. (TIFF 155 kb)

## Abbreviations

BI: Balloon injury
EC: Endothelial cell
Flk-1: Vascular endothelial growth factor receptor 1
GSK3: Glycogen synthase kinase 3
iMDFEC: Induced mesenchymal stem cell-derived functional endothelial cell
MDFEC: Mesenchymal stem cell-derived functional endothelial cell
MSC: Mesenchymal stem cell
PCA: Principal component analysis
SB: SB216763
VCAM-1: Vascular cell adhesion molecule 1
VE-cadherin: Vascular endothelial cadherin
VEGF: Vascular endothelial growth factor
VSMC: Vascular smooth muscle cell
vWF: Von Willebrand factor

## References

[1] Marx SO, Totary-Jain H, Marks AR (2011). Vascular smooth muscle cell proliferation in restenosis. Circ Cardiovasc Interv.

[2] Jukema JW, Verschuren JJ, Ahmed TA, Quax PH (2012). Restenosis after PCI. Part 1: pathophysiology and risk factors. Nat Rev Cardiol.

[3] Curcio A, Torella D, Indolfi C (2011). Mechanisms of smooth muscle cell proliferation and endothelial regeneration after vascular injury and stenting: approach to therapy. Circ J.

[4] Luscher TF, Tanner FC, Tschudi MR, Noll G (1993). Endothelial dysfunction in coronary artery disease. Annu Rev Med.

[5] Asahara T, Bauters C, Pastore C, Kearney M, Rossow S, Bunting S (1995). Local delivery of vascular endothelial growth factor accelerates reendothelialization and attenuates intimal hyperplasia in balloon-injured rat carotid artery. Circulation.

[6] Tahir H, Bona-Casas C, Hoekstra AG (2013). Modelling the effect of a functional endothelium on the development of in-stent restenosis. PLoS One.

[7] Pittenger MF, Mackay AM, Beck SC, Jaiswal RK, Douglas R, Mosca JD (1999). Multilineage potential of adult human mesenchymal stem cells. Science.

[8] Rafii S, Shapiro F, Rimarachin J, Nachman RL, Ferris B, Weksler B (1994). Isolation and characterization of human bone marrow microvascular endothelial cells: hematopoietic progenitor cell adhesion. Blood.

[9] Mead LE, Prater D, Yoder MC, Ingram DA (2008). Isolation and characterization of endothelial progenitor cells from human blood. Curr Protoc Stem Cell Biol.

[10] Zuk PA, Zhu M, Mizuno H, Huang J, Futrell JW, Katz AJ (2001). Multilineage cells from human adipose tissue: implications for cell-based therapies. Tissue Eng.

[11] Chen L, Tredget EE, Liu C, Wu Y (2009). Analysis of allogenicity of mesenchymal stem cells in engraftment and wound healing in mice. PLoS One.

[12] Han Z, Jing Y, Zhang S, Liu Y, Shi Y, Wei L (2012). The role of immunosuppression of mesenchymal stem cells in tissue repair and tumor growth. Cell Biosci.

[13] Wei X, Yang X, Han ZP, Qu FF, Shao L, Shi YF (2013). Mesenchymal stem cells: a new trend for cell therapy. Acta Pharmacol Sin.

[14] Liu JW, Dunoyer-Geindre S, Serre-Beinier V, Mai G, Lambert JF, Fish RJ (2007). Characterization of endothelial-like cells derived from human mesenchymal stem cells. J Thromb Haemost.

[15] Oswald J, Boxberger S, Jorgensen B, Feldmann S, Ehninger G, Bornhauser M (2004). Mesenchymal stem cells can be differentiated into endothelial cells in vitro. Stem Cells.

[16] Silva GV, Litovsky S, Assad JA, Sousa AL, Martin BJ, Vela D (2005). Mesenchymal stem cells differentiate into an endothelial phenotype, enhance vascular density, and improve heart function in a canine chronic ischemia model. Circulation.

[17] Janeczek Portalska K, Leferink A, Groen N, Fernandes H, Moroni L, van Blitterswijk C (2012). Endothelial differentiation of mesenchymal stromal cells. PLoS One.

[18] Ding S, Wu TY, Brinker A, Peters EC, Hur W, Gray NS (2003). Synthetic small molecules that control stem cell fate. Proc Natl Acad Sci U S A.

[19] Hwang KC, Kim JY, Chang W, Kim DS, Lim S, Kang SM (2008). Chemicals that modulate stem cell differentiation. Proc Natl Acad Sci U S A.

[20] Song H, Hwang HJ, Chang W, Song BW, Cha MJ, Kim IK (2011). Cardiomyocytes from phorbol myristate acetate-activated mesenchymal stem cells restore electromechanical function in infarcted rat hearts. Proc Natl Acad Sci U S A.

[21] Song H, Chang W, Song BW, Hwang KC (2012). Specific differentiation of mesenchymal stem cells by small molecules. Am J Stem Cells.

[22] Song H, Chang W, Lim S, Seo HS, Shim CY, Park S (2007). Tissue transglutaminase is essential for integrin-mediated survival of bone marrow-derived mesenchymal stem cells. Stem Cells.

[23] Zhang L, McCabe T, Condra JH, Ni YG, Peterson LB, Wang W (2012). An anti-PCSK9 antibody reduces LDL-cholesterol on top of a statin and suppresses hepatocyte SREBP-regulated genes. Int J Biol Sci.

[24] Vestweber D (2008). VE-cadherin: the major endothelial adhesion molecule controlling cellular junctions and blood vessel formation. Arterioscler Thromb Vasc Biol.

[25] Garmy-Susini B, Jin H, Zhu Y, Sung RJ, Hwang R, Varner J (2005). Integrin alpha4beta1-VCAM-1-mediated adhesion between endothelial and mural cells is required for blood vessel maturation. J Clin Invest.

[26] Blaise GA, Stewart DJ, Guerard MJ (1993). Acetylcholine stimulates release of endothelium-derived relaxing factor in coronary arteries of human organ donors. Can J Cardiol.

[27] Asahara T, Chen D, Tsurumi Y, Kearney M, Rossow S, Passeri J (1996). Accelerated restitution of endothelial integrity and endothelium-dependent function after phVEGF165 gene transfer. Circulation.

[28] Fujiyama S, Amano K, Uehira K, Yoshida M, Nishiwaki Y, Nozawa Y (2003). Bone marrow monocyte lineage cells adhere on injured endothelium in a monocyte chemoattractant protein-1-dependent manner and accelerate reendothelialization as endothelial progenitor cells. Circ Res.

[29] Gulati R, Jevremovic D, Peterson TE, Witt TA, Kleppe LS, Mueske CS (2003). Autologous culture-modified mononuclear cells confer vascular protection after arterial injury. Circulation.

[30] Coghlan MP, Culbert AA, Cross DA, Corcoran SL, Yates JW, Pearce NJ (2000). Selective small molecule inhibitors of glycogen synthase kinase-3 modulate glycogen metabolism and gene transcription. Chem Biol.

[31] Liu C, Li Y, Semenov M, Han C, Baeg GH, Tan Y (2002). Control of beta-catenin phosphorylation/degradation by a dual-kinase mechanism. Cell.

[32] Aberle H, Bauer A, Stappert J, Kispert A, Kemler R (1997). beta-catenin is a target for the ubiquitin-proteasome pathway. EMBO J.

[33] Li VS, Ng SS, Boersema PJ, Low TY, Karthaus WR, Gerlach JP (2012). Wnt signaling through inhibition of beta-catenin degradation in an intact Axin1 complex. Cell.

[34] Grimes CA, Jope RS (2001). The multifaceted roles of glycogen synthase kinase 3beta in cellular signaling. Prog Neurobiol.

[35] Meijer L, Flajolet M, Greengard P (2004). Pharmacological inhibitors of glycogen synthase kinase 3. Trends Pharmacol Sci.

[36] Bai K, Huang Y, Jia X, Fan Y, Wang W (2010). Endothelium oriented differentiation of bone marrow mesenchymal stem cells under chemical and mechanical stimulations. J Biomech.

[37] Whyte JL, Ball SG, Shuttleworth CA, Brennan K, Kielty CM (2011). Density of human bone marrow stromal cells regulates commitment to vascular lineages. Stem Cell Res.

[38] Han L, Shao J, Su L, Gao J, Wang S, Zhang Y (2012). A chemical small molecule induces mouse embryonic stem cell differentiation into functional vascular endothelial cells via Hmbox1. Stem Cells Dev.

[39] Lian X, Bao X, Al-Ahmad A, Liu J, Wu Y, Dong W (2014). Efficient differentiation of human pluripotent stem cells to endothelial progenitors via small-molecule activation of WNT signaling. Stem Cell Reports.

