# Supplementary figures and images for: 1H-pyrrole-2,5-dione-based small molecule-induced generation of mesenchymal stem cell-derived functional endothelial cells that facilitate rapid endothelialization after vascular injury
Source: Stem Cell Res Ther. 2015 Sep 15;6(1):174. doi: 10.1186/s13287-015-0170-6 (PMC4572653; doi:10.1186/s13287-015-0170-6)

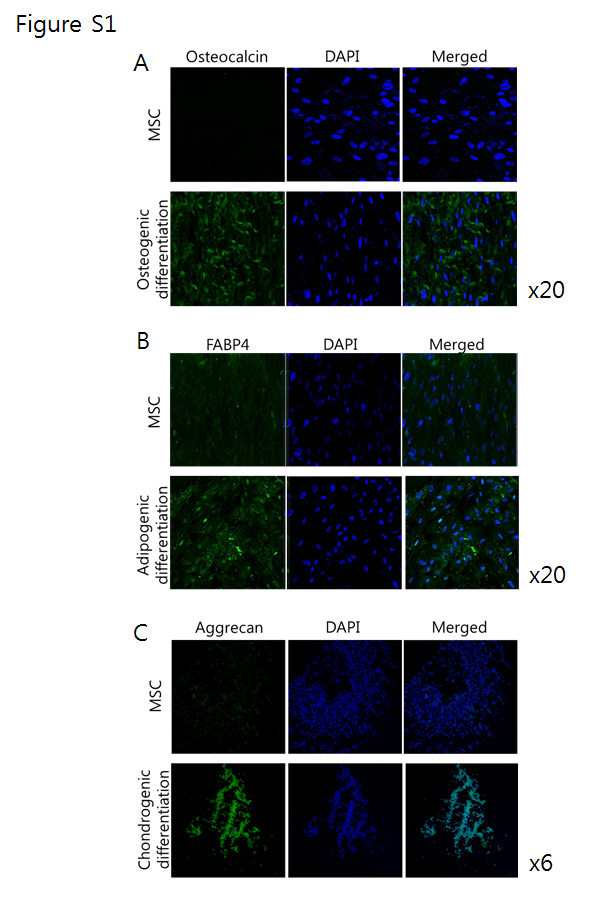

Supplement: Additional file 1: Figure S1. — Differentiation potential of rat MSCs isolated by using an in-house protocol. (A) Osteogenic differentiation of MSCs was detected by immunocytochemistry by using anti-osteocalcin antibodies. (B) Adipogenic differentiation of MSCs was detected by immunocytochemistry by using anti-FABP4 (fatty acid-binding protein 4) antibodies. (C) Chondrogenic differentiation of MSCs was detected by immunocytochemistry by using anti-aggrecan antibodies. MSC mesenchymal stem cell. (TIFF 418 kb) [file 13287_2015_170_MOESM1_ESM.tiff]

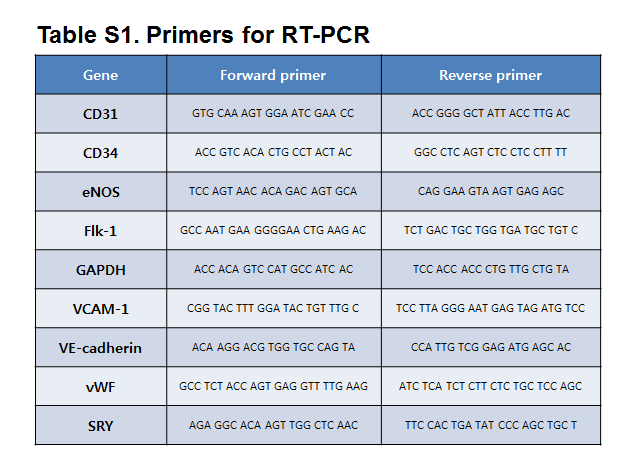

Supplement: Additional file 2: Table S1. — Primers for reverse transcription-polymerase chain reaction. (TIFF 62 kb) [file 13287_2015_170_MOESM2_ESM.tiff]

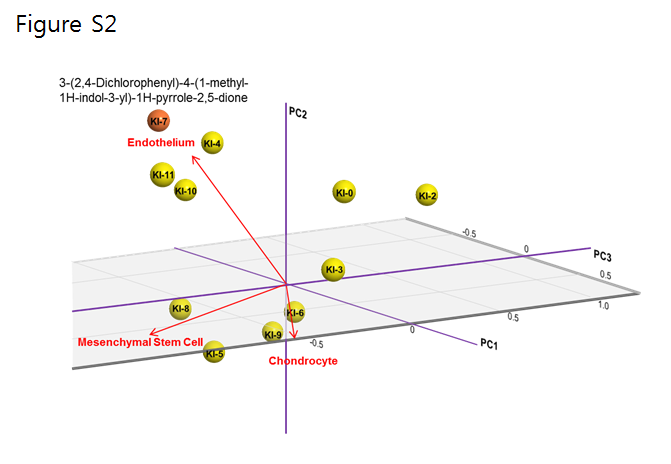

Supplement: Additional file 3: Figure S2. — Principal component analysis (PCA) suggests that the small molecule KI-7 will induce the EC differentiation of MSCs. The PCA results showing a cross-relationship between specific cell types and the screened small molecules (yellow balls). Chemical names with targets in parenthesis: KI-0: no inhibitor; KI-1: lavendustin (5-(N-2,5-dihydroxybenzyl) aminosalicylic acid (CaMKII); KI-2:(4-(4-(2,3-dihydrobenzo [1, 4] dioxin-6-yl)-5-pyridin-2-yl-1H-imidazol-2-yl)benzamide (CKI); KI-3: 6-cyclohexylmethoxy-2-(4-sulfamoylanilino) purine (CDK1,2); KI-4: 3-(pyridin-2-yl)-4-(4-quinonyl)]-1Hpyrazole (TGFβRI kinase); KI-5: N-[2-((p-bromocinnamyl) amino)ethyl]-5-isoquinolinesulfonamide, 2HCl (PKA); KI-6: (2-[1-(3-dimethylaminopropyl)-5-methoxyindol-3-yl]-3-(1H-indol-3-yl)) maleimide (PKC); KI-7: 3-(2,4-dichlorophenyl)-4-(1-methyl-1H-indol-3-yl)-1H-pyrrole-2,5-dione (GSK3β); KI-8: 4-[(3-bromophenyl) amino]-6,7-diethoxyquinazoline (PTK); KI-9: N- (4-pyridyl)-N-(2,4,6-trichlorophenyl) urea (ROCK); KI-10: 4,5-dimethoxy-2-nitrobenzaldehyde (DNA-PK); KI-11: 4-(4-fluorophenyl)-2-(4-hydroxyphenyl)-5-(4-pyridyl)1H-imidazole (p38 MAPK). CaMK calcium/calmodulin-dependent protein kinase; CDK cyclin-dependent kinase, CK casein kinase I, DNA-PK DNA-dependent protein kinase, GSK glycogen synthase kinase, KI kinase inhibitor, MAPK mitogen-activated protein kinase, MSC mesenchymal stem cell, PC principal component, PKA protein kinase A, PKC protein kinase C, PTK protein tyrosine kinase, ROCK rho-associated protein kinase, TGFβ RI transforming growth factor beta type I receptor. (TIFF 91 kb) [file 13287_2015_170_MOESM3_ESM.tiff]

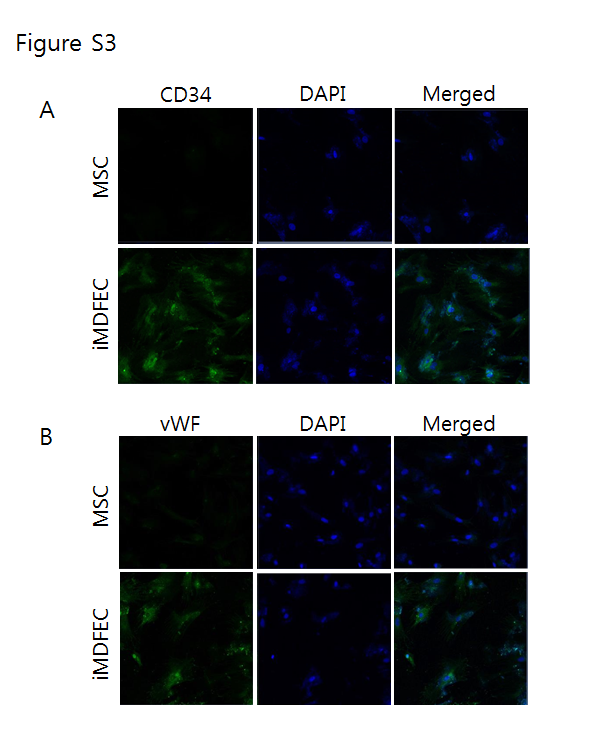

Supplement: Additional file 4: Figure S3. — EC marker expression in iMDFECs. MSCs treated with SB for 16 days to induce EC differentiation. (A) CD34 expression was measured by immunocytochemistry by using anti-CD34 antibodies. (B) vWF expression was measured by immunocytochemistry by using anti-vWF antibodies. EC endothelial cell, iMDFEC induced mesenchymal stem cell-derived functional endothelial cell, MSC mesenchymal stem cell, SB SB216763, vWF von Willebrand factor. (TIFF 269 kb) [file 13287_2015_170_MOESM4_ESM.tiff]

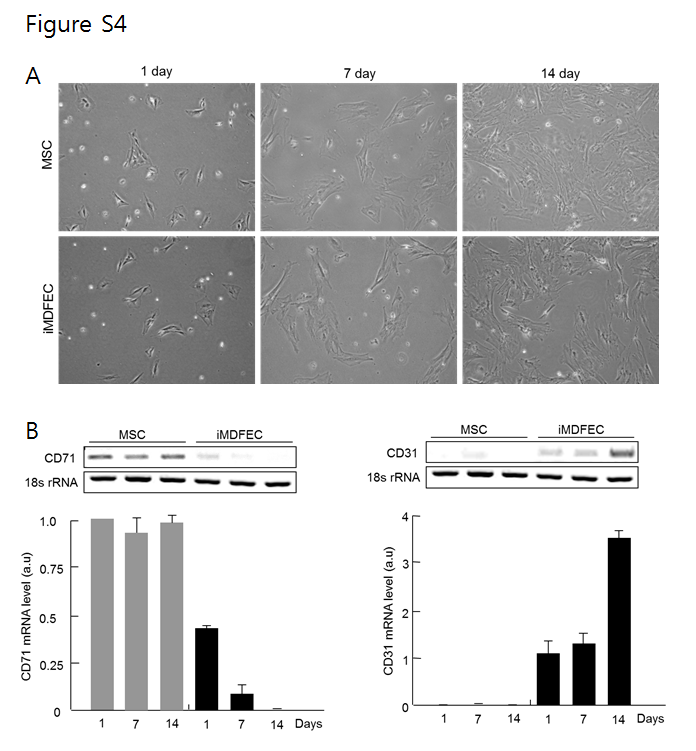

Supplement: Additional file 5: Figure S4. — Time-dependent changes of iMDFECs. (A) Morphological examination of iMDFECs at indicated time points. (B) The mRNA expression levels of the MSC marker CD71 and the EC marker CD31 were examined at the indicated time points. Data represent the mean ± standard deviation of at least three independent experiments. EC endothelial cell, iMDFEC induced mesenchymal stem cell-derived functional endothelial cell, MSC mesenchymal stem cell. (TIFF 270 kb) [file 13287_2015_170_MOESM5_ESM.tiff]

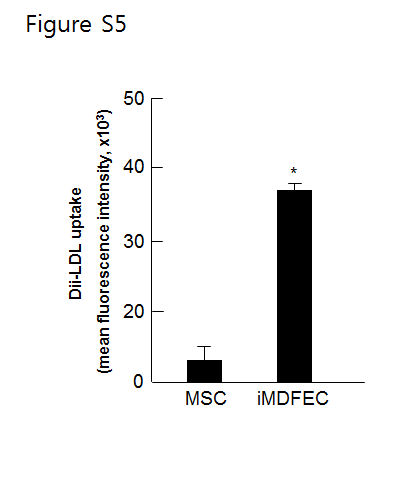

Supplement: Additional file 6: Figure S5. — Lipid uptake assay using DiI-LDL. MSCs or iMDFECs were incubated with DiI-LDL (10 μg/ml) for 4 h. The cells were lysed in 0.1 N NaOH and 0.1 % SDS, and the amount of DiI-LDL was determined by fluorescence reading (excitation/emission at 530/580 nm). The fluorescence of DiI-LDL was normalized by the cell lysate protein concentrations. DiI-LDL 3,3′-dioctadecylindocarbocyanine-low density lipoprotein, iMDFEC induced mesenchymal stem cell-derived functional endothelial cell, MSC mesenchymal stem cell. (TIFF 33 kb) [file 13287_2015_170_MOESM6_ESM.tiff]

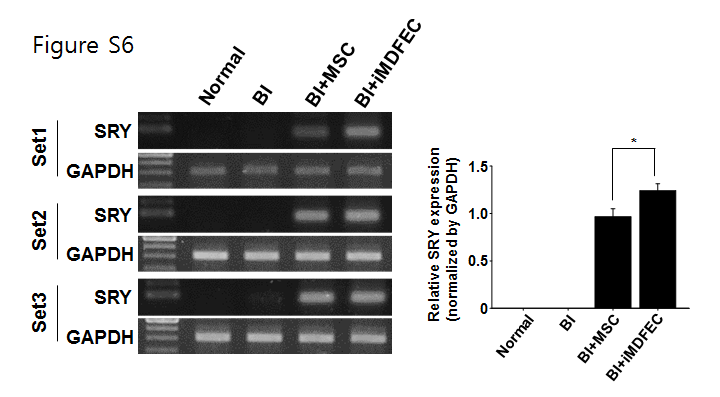

Supplement: Additional file 7: Figure S6. — Evaluation of amount of MSCs or iMDFECs incorporated by using SRY as a marker of incorporated male-origin transplanted cells. Total RNA was prepared from the common carotid artery harvested at day 21 after the balloon injury. Data represent the mean ± standard deviation of three independent experiments. iMDFEC induced mesenchymal stem cell-derived functional endothelial cell, MSC mesenchymal stem cell. (TIFF 114 kb) [file 13287_2015_170_MOESM7_ESM.tiff]

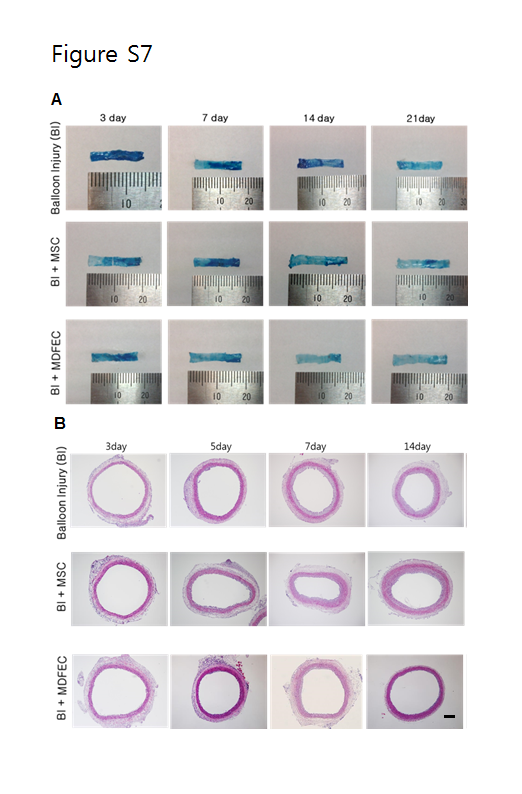

Supplement: Additional file 8: Figure S7. — Time-dependent changes in vessel permeability and neointima thickness after transplantation of MSCs/MDFECs in balloon-injured animals. (A) Images of an Evans Blue-stained carotid artery showing transmural coverage. More blue indicates increased permeability. (B) Images of neointima formation at different time points. Scale bar = 200 μm. MDFEC mesenchymal stem cell-derived functional endothelial cell, MSC mesenchymal stem cell. (TIFF 369 kb) [file 13287_2015_170_MOESM8_ESM.tiff]

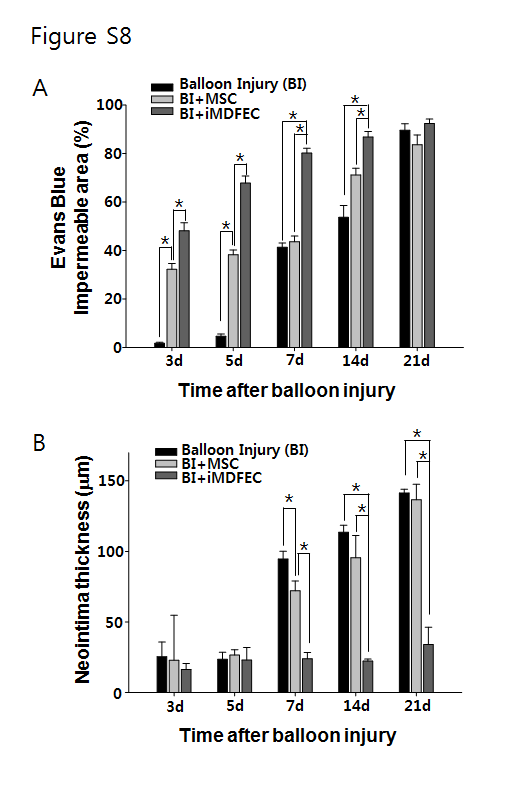

Supplement: Additional file 9: Figure S8. — Quantification of time-dependent changes in neointima thickness and vessel permeability after transplantation of MSCs/iMDFECs in balloon-injured animals. (A) Time-dependent changes in the Evans Blue-impermeable area after balloon injury were measured. (B) Time-dependent changes in the neointima thickness after balloon injury were evaluated. *P < 0.05. iMDFEC induced mesenchymal stem cell-derived functional endothelial cell, MSC mesenchymal stem cell. (TIFF 73 kb) [file 13287_2015_170_MOESM9_ESM.tiff]

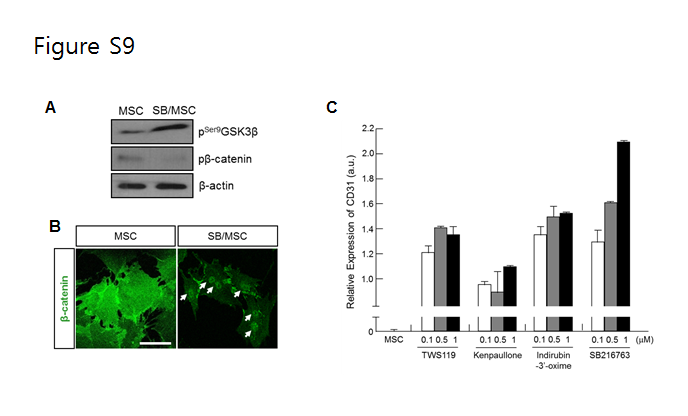

Supplement: Additional file 10: Figure S9. — Effect of SB on β-catenin activation and EC differentiation and efficacy of different GSK3β inhibitors. (A) The expression levels of phosphorylated GSK3β (pser9GSK3β) and β-catenin (pβ-catenin) and (B) the nuclear localization of β-catenin were examined after MSCs were treated with 1 μM SB216763 for 24 h. Scale bar = 50 μm. White arrows indicate nuclear translocated β-catenin. (C) Effect of different GSK3β inhibitors (TWS119, kenpaullone, and indirubin-3′-oxime) on the CD31 induction of MSCs. MSCs were treated with different GSK3β inhibitors with varying concentrations (0.1, 0.5, and 1 μM) for 16 days, and the expression of CD31 was examined. EC endothelial cell, GSK3β glycogen synthase kinase 3 beta, MSC mesenchymal stem cell, SB SB216763. (TIFF 112 kb) [file 13287_2015_170_MOESM10_ESM.tiff]

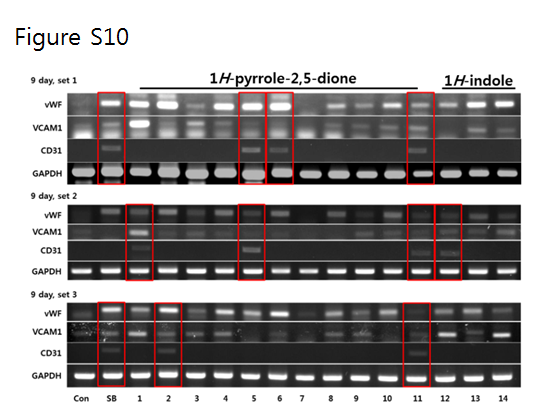

Supplement: Additional file 11: Figure S10. — 1H-pyrrole-2,5-dione moiety is important to induce EC-like differentiation of MSCs. Screening of SB216763 derivatives for inducing EC marker expressions was conducted. To examine the potency of SB216763 derivatives for inducing EC differentiation of MSCs, the MSCs were treated 1 μM of each derivatives for 9 days (media containing corresponding small molecules were changed every 3 days), and the expressions of EC markers CD31, VCAM1, and vWF were examined by reverse transcription-polymerase chain reaction. Samples were collected at 9 days after the initial treatment. EC endothelial cell, MSC mesenchymal stem cell, VCAM-1 vascular cell adhesion molecule 1, vWF von Willebrand factor. (TIFF 155 kb) [file 13287_2015_170_MOESM11_ESM.tiff]
